# Investigating the Role of the Host Multidrug Resistance Associated Protein Transporter Family in *Burkholderia cepacia* Complex Pathogenicity Using a *Caenorhabditis elegans* Infection Model

**DOI:** 10.1371/journal.pone.0142883

**Published:** 2015-11-20

**Authors:** Pietro Tedesco, Marco Visone, Ermenegilda Parrilli, Maria Luisa Tutino, Elena Perrin, Isabel Maida, Renato Fani, Francesco Ballestriero, Radleigh Santos, Clemencia Pinilla, Elia Di Schiavi, George Tegos, Donatella de Pascale

**Affiliations:** 1 Institute of Protein Biochemistry, National Research Council, Via P. Castellino 111, I-80131, Naples, Italy; 2 Department of Chemical Sciences and School of Biotechnological Sciences, University of Naples Federico II, Via Cintia, I-80126, Naples, Italy; 3 Laboratory of Microbial and Molecular Evolution, Department of Biology, University of Florence, Via Madonna del Piano, I-50019, Sesto Fiorentino, Florence, Italy; 4 School of Biotechnology and Biomolecular Sciences and Centre for Marine Bio-Innovation, University of New South Wales, Sydney, 2052, New South Wales, Australia; 5 Torrey Pines Institute of Molecular Studies, Port St. Lucie, FL, United States of America, and San Diego, CA, United States of America; 6 Institute of Bioscience and BioResources, National Research Council, via P. Castellino 111, I-80131, Naples, Italy; 7 Institute of Genetics and Biophysics, National Research Council, via P. Castellino 111, I-80131, Naples, Italy; 8 Wellman Centre for Photomedicine, Massachusetts General Hospital, Boston, MA, United States of America; 9 Department of Dermatology, Harvard Medical School, Boston, MA, United States of America; Inserm U869, FRANCE

## Abstract

This study investigated the relationship between host efflux system of the non-vertebrate nematode *Caenorhabditis elegans* and *Burkholderia cepacia* complex (Bcc) strain virulence. This is the first comprehensive effort to profile host-transporters within the context of Bcc infection. With this aim, two different toxicity tests were performed: a slow killing assay that monitors mortality of the host by intestinal colonization and a fast killing assay that assesses production of toxins. A Virulence Ranking scheme was defined, that expressed the toxicity of the Bcc panel members, based on the percentage of surviving worms. According to this ranking the 18 Bcc strains were divided in 4 distinct groups. Only the Cystic Fibrosis isolated strains possessed profound nematode killing ability to accumulate in worms’ intestines. For the transporter analysis a complete set of isogenic nematode single Multidrug Resistance associated Protein (MRP) efflux mutants and a number of efflux inhibitors were interrogated in the host toxicity assays. The Bcc pathogenicity profile of the 7 isogenic *C*. *elegans* MRP knock-out strains functionality was classified in two distinct groups. Disabling host transporters enhanced nematode mortality more than 50% in 5 out of 7 mutants when compared to wild type. In particular *mrp-2* was the most susceptible phenotype with increased mortality for 13 out 18 Bcc strains, whereas *mrp-3* and *mrp-4* knock-outs had lower mortality rates, suggesting a different role in toxin-substrate recognition. The use of MRP efflux inhibitors in the assays resulted in substantially increased (>40% on average) mortality of wild-type worms.

## Introduction

The *Burkholderia cepacia* complex (Bcc) occupies a critical position among Gram-negative multi-drug resistant bacteria. It consists of at least 20 closely related species inhabiting different ecological niches, including plants and animals [1–5]. Bcc multi drug and pandrug-resistant opportunistic human pathogens cause problematic lung infections in immune-compromised individuals, including cystic fibrosis (CF) patients [6–8]. Bcc members are naturally resistant to antibiotics including cephalosporins, β-lactams, polymyxins and aminoglycosides, rendering Bcc infections challenging to eradicate [9,10]. There is an imminent need to develop new Bcc antimicrobial therapeutic strategies. Dissecting virulence and pathogenicity determinants as well as identifying novel therapeutic targets may be promising approaches. These tasks may be advanced by the exploitation of the non-vertebrate host models *Drosophila melanogaster*, *Galleria mellonella*, and *Caenorhabditis elegans*. Model hosts have been used to evaluate microbial virulence traits involved in mammalian infections and the efficacy of antimicrobial compounds [11–16]. The free-living nematode *C*. *elegans* is a widespread multicellular organism that is a self-fertilizing hermaphrodite with a rapid generation time. *C*. *elegans* has been proven cost-effective, ethical, reproducible and genetically powerful infection model despite the obvious reported technical limitations (nematodes have lower optimal growth temperatures when compared with human pathogens; occurrence of host specific virulence factors) [15,17–19]. In fact, there is an extensive body of literature for the utility of the nematode to model infection with a variety of Gram-negative bacteria including *Escherichia coli*, *Burkholderia pseudomallei*, *B*. *cepacia* complex and *Pseudomonas aeruginosa* [20–23]. The *C*. *elegans*-Bcc studies in the last decade have shed some light on the complex-nematode interaction, correlating genotypic characteristics of the pathogen with phenotypic changes in the host. These efforts have identified specific virulence factors: the auto inducer dependent Acyl-HomeSerineLactone (*aidA)*, the phenazine biosynthesis regulator *(Pbr)*, and the host factor phage Q, *(hfq*) [16,24–33].

Recent studies have underlined the importance of efflux systems in infection within the content of host-pathogen interaction [34–36]. The host efflux capability is considered part of a basic defence mechanism. For example the *B*. *pseudomallei* infection stimulates the overproduction of the ATP Binding Cassette (ABC) transporter pgp-5 in *C*. *elegans* [37]. However, the partition of host transporters in the infection process has never been studied in depth. Bcc members produce a variety of metabolites and toxins, potential host efflux substrates. Furthermore, exploring the role of host transporters in pathogenicity may facilitate the design of appropriate tools for toxin identification. Multidrug Resistance associated Proteins (MRPs) are members of the ABC efflux transporter family with broad substrate specificity for the transport of endogenous and xenobiotic anionic substances found in Bacteria, Archaea and Eukarya [38–41]. MRPs play important roles in nematode physiology such as control resistance to anthelmintic (ivermectine) and heavy metals (arsenic) [42–44]. This study emphasizes the contribution of the host MRP efflux subfamily to Bcc virulence, employing a panel of 18 strains representing the up-to-date different acknowledged species and a fully functional seven single *C*. *elegans* mutant set impaired in MRPs. A Virulence-Ranking (VR) scheme based on comparing host survival rates in two different assays was developed. This scheme provides the tool for a detailed study on the effect of the MRP transporter family on Bcc virulence using as well as selected efflux inhibitors.

## Materials and Methods

### Bacterial strains, nematode strains and growth conditions

*C*. *elegans* Wild-type (WT) Bristol N2, NL147 (*mrp-1(pk89)* X*)*, RB1713 (*mrp-2(ok2157)* X), RB1028 (*mrp-3(ok955*) X), VC712 (*mrp-4(ok1095*) X), VC1599 (*mrp-5(ok2067)/szT1* X), RB1070 (*mrp-6(ok1027)* X) and RB1269 (*mrp-8(ok1360)* III) strains were obtained from the *Caenorhabditis* Genetic Centre (CGC). For strain VC1599, due to *ok2067* mutation lethality in homozygosis, all the experiments were performed assaying heterozygotes worms. All mutants presented identical phenotypic traits in respect to WT: normal larval development (eggs reaching adults state in 72 h as indicated on standard table (www.wormbook.org)), non-impaired reproduction, and survival rate at 100% when fed with *E*. *coli*. Mutant mpr-5 in heterozygosis also aligned to those parameters. All strains were recovered from frozen stocks, and routinely kept on NGM (Nematode Growth Medium) plates seeded with *E*. *coli* OP50 as a food source [45]. The panel of Bcc strains used in this work belongs to the Bcc collection at the University of Gent, Belgium, and is listed in **Table 1**. Bcc and *E*. *coli* OP50 cells were routinely grown in Luria-Bertani broth (LB) (10 g/L Bacto-tryptone, 5 g/L Yeast extract, 10 g/L NaCl) at 37°C.

**Table 1:** *Burkholderia cepacia* complex used in this work and VR relative to the different killing assays.

Species	Strain	Source	SKA	FKA
***Burkholderia cepacia***	LMG 1222	Onion	**0**	**3**
***Burkholderia multivorans***	LMG 13010	CF	**0**	**0**
***Burkholderia cenocepacia***	LMG 16656	CF	**2**	**2**
***Burkholderia stabilis***	LMG 14294	CF	**3**	**3**
***Burkholderia vietnamiensis***	LMG 10929	Soil	**1**	**0**
***Burkholderia dolosa***	LMG 18943	CF	**0**	**1**
***Burkholderia ambifaria***	LMG 19182	Soil	**0**	**3**
***Burkholderia anthina***	LMG 20980	Soil	**2**	**1**
***Burkholderia pyrrocinia***	LMG 14191	Soil	**0**	**2**
***Burkholderia ubonensis***	LMG 20358	Soil	**2**	**1**
***Burkholderia latens***	LMG 24064	CF	**1**	**1**
***Burkholderia diffusa***	LMG 24065	CF	**2**	**2**
***Burkholderia arboris***	LMG 24066	Soil	**1**	**1**
***Burkholderia seminalis***	LMG 24067	CF	**2**	**2**
***Burkholderia metallica***	LMG 24068	CF	**3**	**3**
***Burkholderia lata***	LMG 22485	Soil	**0**	**1**
***Burkholderia contaminans***	LMG 23361	AI	**1**	**3**
***Burkholderia pseudomultivorans***	LMG 26883	CF	**0**	**1**

Abbreviations: Soil = Soil rhizosphere, AI = Animal Infections, CF = Cystic Fibrosis patients

VRs

0 = 100% > Survival worms > 80%

1 = 79% > Survival worms > 50%

2 = 49% > Survival worms > 6%

3 = 5% > Survival worms > 0%

### Nematode Toxicity Assays

Slow Killing Assay (SKA) was performed against the *C*. *elegans* WT strain N and MRP-mutants. 2.5-cm-diameter plates containing 3 ml of NGM agar (Peptone 2.5 g/L, NaCl 2,9 g/L, Bacto-Agar 17 g/L, CaCl_2_ 1 mM, Cholesterol 5 μg/mL, KH_2_PO_4_ 25 mM, MgSO_4_ 1 mM) were seeded with 50 μl of the overnight Bcc cultures, normalized to an OD_600_, of 1.7 and incubated for 24 h at 37**°**C to allow the formation of a bacterial lawn. This was the standard bacterial growth condition unless otherwise stated. *C*. *elegans* WT strain and MRP-mutants were synchronized by bleaching treatment [46], and 30–40 worms at larval stage 4 (L4), were transferred to each plate and incubated at 20°C for three days. The plates were scored for living worms every 24 h.

Fast Killing assay (FKA) was carried out in 2.5-cm-diameter plates containing 3 ml of Peptone Glucose Sorbitol (PGS) agar medium [25] (Peptone 12 g/L, Glucose 12 g/L, Sorbitol 27.25 g/L, NaCl 12 g/L, Bacto-Agar 17 g/L, CaCl_2_ 1 mM, Cholesterol 5 μg/mL, KH_2_PO_4_ 25 mM, MgSO_4_ 1 mM). Plates were prepared as described above for the SKA. Then, L4 worms from WT strain and MRP-mutants were collected from NGM plates, washed with M9 medium (Na_2_HPO_4_ **·**7H_2_O 12.8 g/L, Na_2_HPO_4_ (anhydrous) 6 g/L, KH_2_PO_4_ 3 g/L, NaCl 0.5 g/L, NH_4_Cl 1 g/L) and 30–40 L4 worms were spotted onto the bacterial lawn. The plates were then incubated at 20°C and scored for living worms every 24 h. In both assays, *E*. *coli* OP50 was used as a negative control. A worm was considered dead when it no longer responded to touch. For statistical purposes, 5 replicates per trial were carried out with a unique egg preparation. The incubation time was set at 2 days. A pathogenicity scheme (VR) was established by comparing the "infectivity" towards nematodes between *E*. *coli* OP50 and Bcc isolates.

### Microscopy analysis

40–60 WT L4 worms were grown on NGM plates seeded with Bcc or *E*. *coli* OP50 propagated in standard growth conditions. Plates were incubated at 20°C, and after 4 and 24 h, the nematodes were inspected using a Zeiss Axioskop microscope equipped with Differential Interference Contrast (DIC) employing 10x, 20x, 40x, 63x and 100x objectives and 10X eyepiece. Images were collected with a Zeiss Axiocam MR digital camera.

### Toxin Diffusion assay

Bcc or *E*. *coli* OP50 cells were grown under standard growth conditions and spread on sterile 0.22 μm Millipore Nitrocellulose (Darmstadt, Germany) filter disk located onto 2.5-cm-diameter PGS plates [25]. After overnight incubation at 37°C, the filter together with the bacterial lawn was removed and the plates were allowed to cool to room temperature. 30–40 hypochlorite-synchronised WT L4 nematodes were spotted onto the conditioned agar. Paralysation and mortality of the worms were detected at 4 and 24 h. The experiments were performed in triplicate, and data reported are mean values ± SD.

### Statistical analysis and clustering

All the Kaplan-Meier survival curves were analyzed using the Graph-pad Prism 5 software. Comparisons *vs*. control for both the *C*. *elegans* and inhibitor experiments were performed using Fisher’s exact test to account for possible non-Normality in the data. In particular, as it was observed that replicate means of *C*. *elegans* percent mortality correlated extremely well to pooled percent mortality (R^2^ > 0.99 in all cases), counts of *C*. *elegans* that were alive and dead after 72 h were used to populate the various 2x2 tables onto which the Fisher’s exact test was applied. Bonferroni-Holm correction of p-values was used to account for the multiple comparisons performed.

Mutant clustering analysis was performed using hierarchical clustering via Ward’s method. Clusters were fixed using a consistency threshold of 1.1, resulting in cophenetic coefficient (correlation between cluster and metric distance) of at least 0.80.

### Transporter Inhibitor assays

The MRP transporter inhibitors mometasone furoate, lasalocid A sodium, verapamil hydrochloride were purchased from Sigma-Aldrich, Saint Louis, MO. Compounds were dissolved in DMSO and spread onto NGM plates in different concentration ranges: 25–100 μM (mometasone and verapamil) and 125–500 nM (lasalocid). DMSO (0,5% w/v) was used as control. Subsequently, Bcc strains (grown in standard conditions) were spotted onto the plates that were incubated overnight at 37°C. After the incubation 30–40 WT L4 worms were spotted onto the bacterial lawn. The plates were then incubated at 20°C for 3 days and scored for living worms every 24 h. The experiments were performed in triplicate, and the data reported are mean values.

## Results and Discussion

### Killing of *C*. *elegans* by Bcc strains

**Fig 1:**
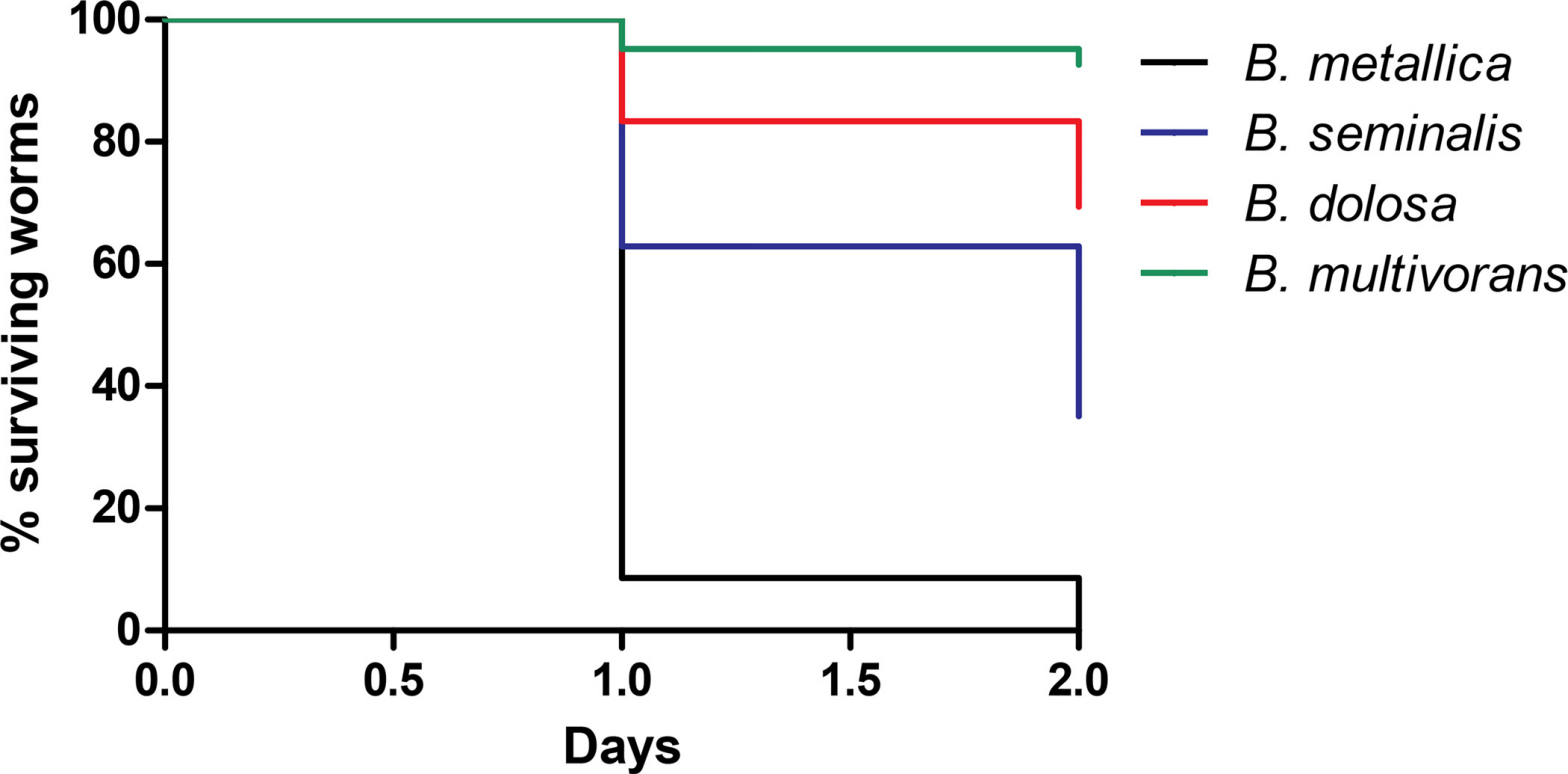
Kaplan-Meier survival plots for L4 N2 worms fed with exemplifying Bcc strains for different VR grown on PGS medium. Worms fed on: B. metallica (VR 3; black line; n = 113; 0% survival at day 2); *B*. *seminalis* (VR 2; blue line; n = 150; 34% survival at day 2); *B*. *dolosa* (VR 1; redline; n = 198; 69% survival at day 2); *B*. *multivorans* (VR 0; green line; n = 120; 93% survival worms). n: Number of worms at day 0. All p-values, comparing each survival curve between them, resulted to be < 0.0001, calculated with "Log-rank (Mantel-Cox) Test" with the Graph-pad Prism 5 software.

To evaluate Bcc virulence determinants and properties, two different assays were performed: i) SKA, performed on a low osmolarity medium (NGM), assigned to correlate worms mortality with intestinal bacterial accumulation/colonisation [24,25]; ii) FKA carried out on a high osmolarity medium (PGS) to demonstrate the secretion of bacterial toxins and evaluate their capacity to paralyse and kill the nematodes [24,25]. A VR was established for the Bcc strains under investigation by comparing the "infectivity" against nematodes between *E*. *coli* OP50 and Bcc isolates. The VR ranges from 0 to 3 (see **Fig 1**) and was based on the percentage of surviving worms after the period of observation, which was set at 3 days. A Bcc strain was considered to be non-pathogenic (VR = 0) when no symptom of disease was observed during the course of nematodes infection and the percentage of live worms at the conclusion of the period of observation ranged from 100 to 80%; VR = 1 corresponded to a percentage of alive worms between 79 to 50%; VR = 2 corresponded to a percentage of alive worms between 49 to 6%; finally, the VR was considered 3 when the percentage of surviving worms was ≤ 5%.

SKA performed against WT L4 worms revealed diverse pathogenicity capabilities among the 18 Bcc representatives **[Table 1]**:

- 2 Bcc strains (*B*. *metallica*, *B*. *stabilis*) displayed high nematocide activity (VR 3). No viable nematodes were detectable in the plates after 3 days of incubation at 20°C.
- Half of the Bcc strains exhibited VR between 1 and 2, showing an intermediate toxicity towards *C*. *elegans*.
- Seven Bcc strains (*B*. *ambifaria*, *B*. *cepacia*, *B*. *dolosa*, *B*. *pseudomultivorans*, *B*. *pyrrocinia*, *B*. *lata* and *B*. *multivorans*) were unable to kill worms, and the whole population was viable (VR = 0).

Nematodes killed in the lawn of bacteria took on a ghostly and hollow “shell-like” appearance about 48 h after the L4 were first introduced, and their shells induced by *B*. *ubonensis*, *B*. *metallica* and *B*. *stabilis* were defined as “chalk-mark ghosts”. This shape is characteristic of organisms lacking a discernible internal cell structures. Often the ghosts eroded to a mere outline.

**Fig 2:**
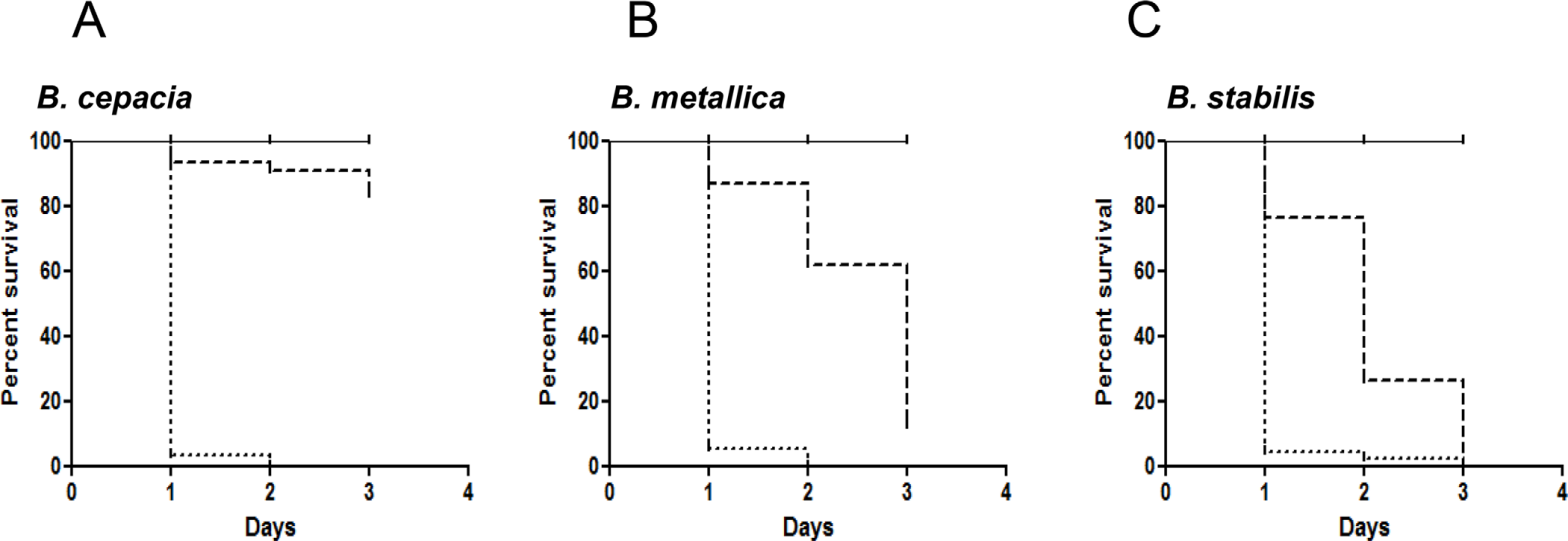
Kaplan-Meier survival plots for L4 stage WT worms fed with: E. coli OP50 (solid lines), Bcc strains on NGM (dashed lines), Bcc strains on PGS (dotted lines). n: Number of worms at day 0. **A)** The pathogenicity of *Bcc* strain *B*. *cepacia* on SKA (n = 93) was compared with the ability on FKA (n = 184). **B)** The pathogenicity of *Bcc* strain *B*. *metallica* on SKA (n = 80) was compared with the ability on FKA (n = 113). **C)** The pathogenicity of *Bcc* strain *B*. *stabilis* on SKA (n = 87) was compared with the ability on FKA (n = 161). P-values were calculated between survival curves on FKA and SKA of each bacteria, and resulted to be < 0.0001 calculated with "Log-rank (Mantel-Cox) Test" with the Graph-pad Prism 5 software.

The pathogenicity of the 18 Bcc strains was then assessed on FKA. Data obtained are summarised in **Table 1**. Nematodes death on FKA appeared to be a rapid process as they loose locomotor functions, as shown by the quick onset of lethargy. Motility visibly decreased after exposure for 4 h, and the rate of foraging was similarly affected in the same time frame. In FKA, five strains (*B*. *ambifaria*, *B*. *cepacia*, *B*. *contaminans*, *B*. *metallica*, *B*. *stabilis*) demonstrated deep killing ability (VR = 3) against *C*. *elegans* and only two strains (*B*. *multivorans* and *B*. *vietnamiensis*) were completely ineffective in killing worms. For the highly active strains, almost 100% mortality occurred in 24 h, while on SKA 3 days are required for complete killing (**Fig 2).**

Nine out of 18 Bcc strains have previously been characterized using SKA [30,47]. The VR of 7 strains (*B*. *anthina*, *B*. *ubonensis*, *B*. *vietnamiensis*, *B*. *cenocepacia B*. *dolosa B*. *ambifaria*, *B*. *cepacia*) are consistent with the previously reported SKA ranking. The same comparison revealed a variation for *B*. *pyrrocinia* and *B*. *stabilis*, which were found as more and less virulent, respectively. This variability may be due to ranking differences, as the experimental conditions were very reproducible. This is the first report for an indicative pathogenicity ranking for 8 *Burkholderia* species, recently added to Bcc, (*B*. *latens*, *B*. *diffusa*, *B*. *arboris*, *B*. *seminalis*, *B*. *metallica*, *B*. *pseudomultivorans*, *B*. *lata*, *B*. *contaminans*). *B*. *metallica*, *B*. *stabilis* (both isolated from CF patients) were the most virulent in both assays (**Fig 2B and 2C**). The comparison of data obtained in the FKA and SKA revealed that, on average, strains isolated from CF patients appeared more virulent than environmental isolates on SKA. In particular, three Bcc strains (*B*. *ambifaria*, *B*. *cepacia* and *B*. *pyrrocinia*) exhibited high nematocide activity in the FKA, whereas they were unable to kill the worms in the SKA (VR 0 and 1). Therefore we can assume that toxin production is a common virulence mechanism for Bcc members, while CF isolates might have acquired different pathogenic traits that allow them to infect and colonize hosts, as already proposed by Pirone et al. [48]. The only exception is represented by *B*. *multivorans*, and *B*. *pseudomultivorans*. These two strains are CF isolates, but were non-virulent towards nematodes. This evidence likely rely in the limitation of the nematode host model, once more indicating that virulence factors are not universal for all hosts [15].

#### Bacterial intestinal accumulation

The two Bcc strains with VR = 3 in the SKA (*B*. *stabilis* and *B*. *metallica*) were then assessed for their ability to accumulate in the *C*. *elegans* intestine. Worms grown in standard condition were inspected using a compound microscope at different incubation times to evaluate the bacterial accumulation in the intestinal lumen.

**Fig 3:**
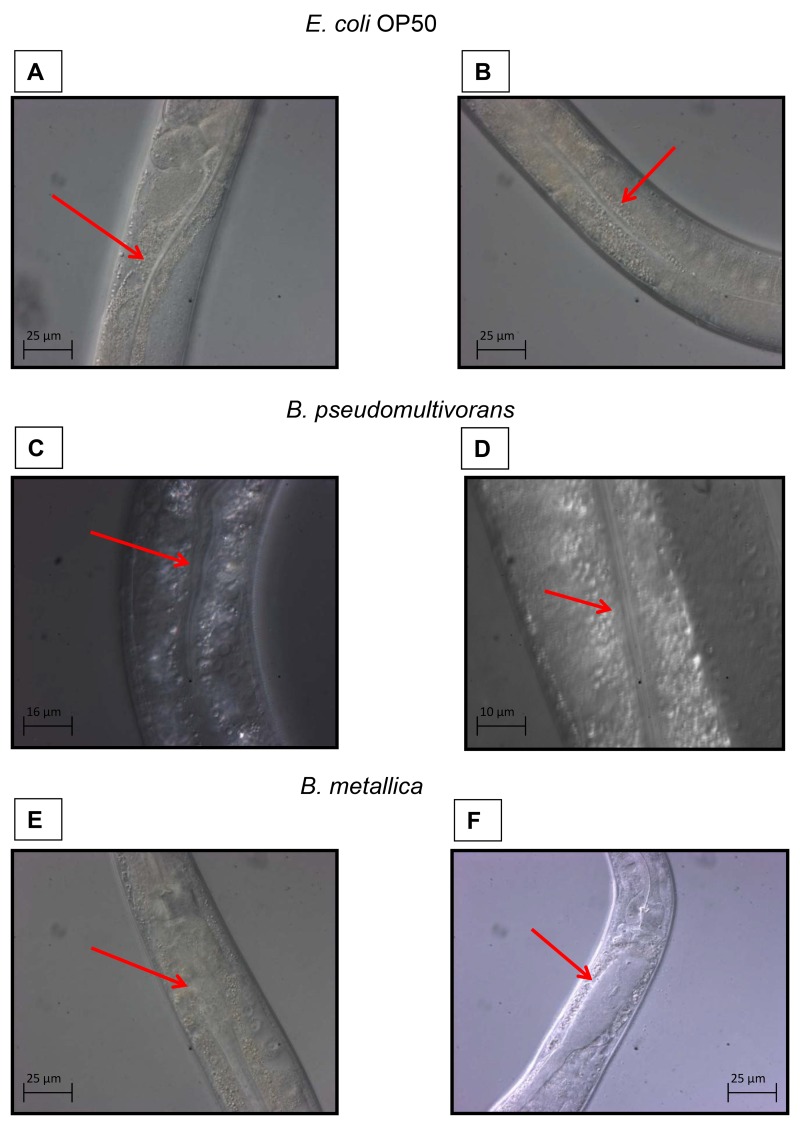
The ability of *Bcc* strains to accumulate in *C*. *elegans* intestinal lumen was evaluated with microscopy analysis. Red arrows indicate the nematodes intestine. **A)** Intestinal lumen of one L4 stage WT worm after 4 h of incubation on NGM plate spotted with *E*. *coli* OP50, and **B)** after 24 h of incubation on the same plate. **C)** Intestinal lumen of one L4 WT after 4 h of incubation on NGM plate spotted with *B*. *pseudomultivorans* (VR 0 on SKA, and **D)** after 24 h of incubation on the same plate. **E)** Intestinal lumen of one L4 WT after 4 h of incubation on NGM plate spotted with *B*. *metallica* (VR 3 on SKA, and **F)** after 24 h of incubation on the same plate.

Bcc colonization of nematode occurred rapidly. After 4 h of incubation, worms fed with *E*. *coli* OP50 showed a thin intestinal lumen **(Fig 3A and 3B),** whereas, when spotted onto *B*. *metallica* layer, worms already presented deformed intestines (**Fig 3E**). After 24 h nematodes displayed a full intestinal lumen packed with bacteria (**Fig 3F).** These data confirmed that Bcc with high VR were able to accumulate within the entire nematode intestine and therefore slow-killing may resemble an infection-like process. On the contrary, nematodes exposed to the strain *B*. *pseudomultivorans*, which exhibited a low pathogenicity (VR = 0 on SKA), under the same experimental conditions presented a healthy intestine with the presence of bacterial cells only in the first part of the intestine (**Fig 3C and 3D**). This finding may signify that: i) even non-pathogenic Bcc strains were able to pass intact through the pharynx and occupy the intestine; ii) the accumulation of the Bcc in the whole nematode gut, especially in the last part of the intestine, might be responsible for the worm’s death [24].

#### Toxin Diffusion assay

**Fig 4:**
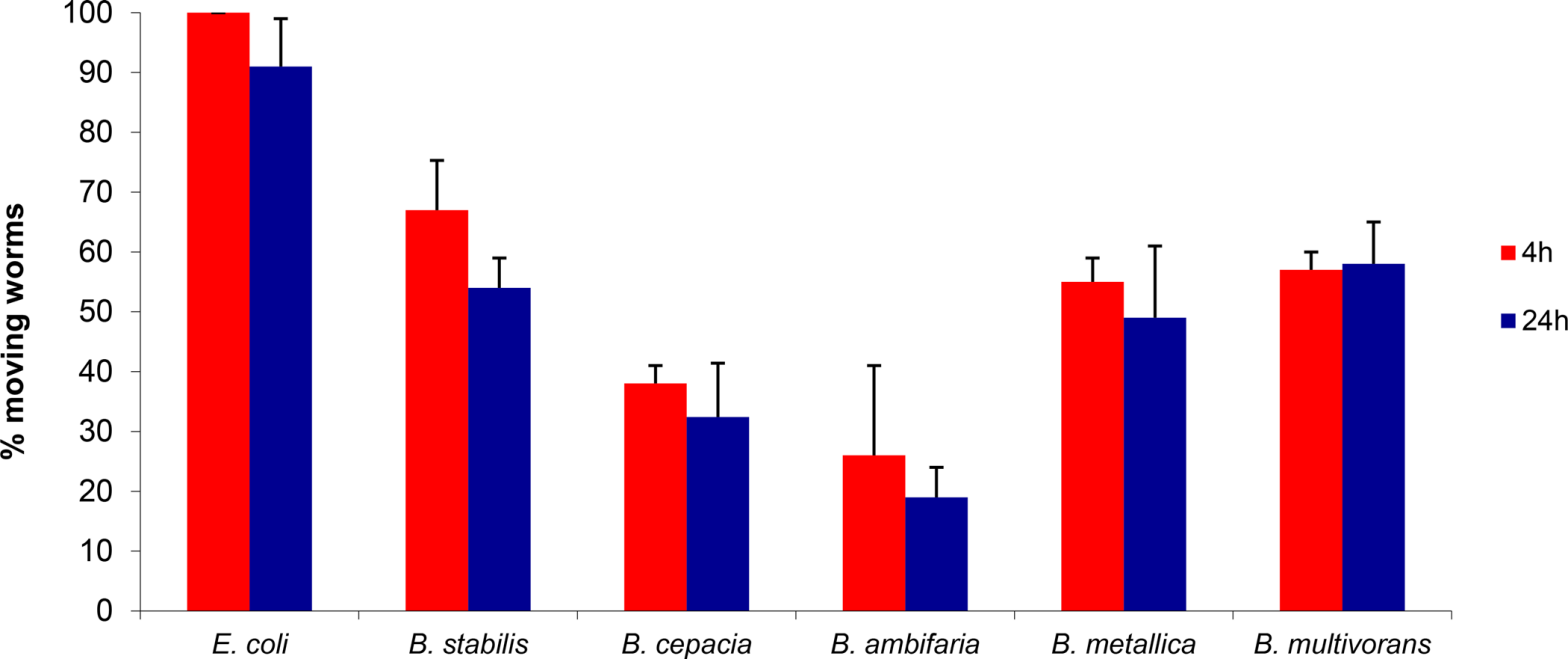
Secreted compounds or toxins mediate fast killing. Data reports paralysis and mortality at 4 and 24 h of worms plated on PGS medium plates treated with Bcc strains or E. coli grown on a sterile disk. Data represent mean values of three independent experiments and SD values are reported. P-values were calculated between sample (Bcc) and control (OP50) at the corresponding time, and were always < 0.05.

To evaluate the contribution of diffusible secreted factors (toxins and/or other virulence chemical signalling molecules) to the rapid kinetics of killing on FKA, we performed the toxin diffusion assay [25]. These experiments were carried out on a reduced panel consisting of the five Bcc strains possessing the highest nematocide activity on FKA (*B*. *contaminans*, *B*. *cepacia*, *B*. *ambifaria*, *B*. *metallica* and *B*. *stabilis*). Results shown in **Fig 4**revealed that a high percentage of worms were paralyzed after 4 h of incubation on plates, even if they were not in contact with the bacteria. In particular, only 30% of the worms placed on *B*. *ambifaria* plates were still mobile and active, whereas the remaining nematode population appeared paralysed. On the contrary, worms spotted on plates containing *E*. *coli* conditioned agar did not present any paralysis or mortality. Among the tested Bcc strains, *B*. *ambifaria* was the most active toxin producer. Indeed, after 24 h of incubation only 20% of the total number of nematodes was still moving on *B*. *ambifaria* plates (**Fig 4)**. In the case of *B*. *stabilis*, it was observed that paralyzed worms at 4 h were able to move again and survive. One plausible explanation for this variation might be related to low stability of the diffusible toxins/virulence determinants produced by those strains that require constant production.

Interestingly, when the toxin filter assay was performed on NGM medium, no paralysis or mortality was detected. This experiment confirms that FKA rapid killing kinetics revealed a role for diffusible toxins as a main component of the infectious process. Thomson and Dennis demonstrated the production by Bcc strains of a haemolytic toxin required for full virulence, synthesized by a non-ribosomal peptide synthase (NRPS) pathway, typical of a complex secondary metabolite [49]. They screened a panel of Bcc strains including *B*. *cenocepacia*, *B*. *stabilis*, *B*. *pyrrocinia* and *B*. *vietnamiensis* for the presence of this gene cluster. A NRPS cluster was identified in *B*. *pyrrocinia* and *B*. *stabilis* with VR = 3 on FKA. Moreover, Bcc strains are known to produce toxins with demonstrated antifungal activity like the cyclic peptides occidiofungins (burkholdines) [50]. Therefore, we cannot *a priori* exclude the possibility that a peptide might represent the toxin active towards *C*. *elegans*.

### Killing of MRPs knock-out *C*. *elegans* mutants by Bcc member strains

The nematode-Bcc pathogenicity ranking system developed was investigated for its ability to detect and map genotype-specific host responses. We obtained access to a complete, seven MRPs knock-out nematode mutant set, *mrp-1(pk89)*, *mrp-2(ok2157)*, *mrp-3(ok955*), *mrp-4(ok1095*), *mrp-5(ok2067)*, *mrp-6(ok1027)* and *mrp-8(ok1360*), impaired in the corresponding ABC membrane transporters.

These knock-out mutants exhibited identical phenotypic attributes with the WT. The 18 Bcc representative strains were profiled against the 7 mutants in both SKA and FKA. Control mortality was calculated to be the number of dead worms divided by the number of total worms. Pooled mortality counts (alive *vs*. dead) for each mutant were tested against the WT using Fisher’s Exact test. Statistically significant (Bonferroni-Holm corrected p-value < 0.05) differences from WT are shown in **Table 2**. The mortality rates calculated were highly variable suggesting a Bcc strain-specific effect towards the MRP *C*. *elegans* mutants. Some trends were detected: *mrp-5* and *mrp-2* had increased mortality rate for several Bcc strains in both SKA and FKA. Specifically, 8 Bcc strains in SKA and 9 in FKA showed increased killing towards *mrp-5*, while 8 strains in SKA and 8 in FKA appeared more virulent against *mrp-2*. *C*. *elegans* mutants *mrp-3* and *mrp-4* displayed lower killing rates when incubated with several Bcc strains. In particular, mutant *mrp*-4 exhibited decreased mortality to 8 Bcc species in SKA and to 7 strains in FKA. Regarding the Bcc strains, on SKA *B*. *ambifaria* displayed increased virulence towards the whole mutant set, with mortality rate compared to the WT higher than 75% and 71% towards, *mrp-5 and mrp-6* respectively. *B*. *arboris* demonstrated an increased pathogenic effect towards *mrp-1*, *mrp-2*, *mrp-6*, *mrp-8*, while *B*. *dolosa* was more lethal against *mrp-2*, *mrp-5*, *mrp-6*, *mrp-8* mutants. On FKA, *B*. *lata* and *B*. *multivorans* were the most pathogenic strains with increased mortality rate against all mutants, while *B*. *diffusa* was more virulent against 6 mutants, and *B*. *arboris* against 5 mutants.

**Table 2:** *MRP knock-out C*. *elegans* mutants mortality expressed as percentage of dead worms and comparison between the mutants and the WT. Mutant mrp-5 was tested as heterozygote, due to lethality of the mutation in homozygosis. Statistical significant differences appear highlighted, with negative values indicating statistically significant reductions in mortality from WT, and with positive values indicating statistically significant increases in mortality from WT.

			Significant Changes in % Mortality					
Strain	WT Mortality FKA	mrp-1	mrp-2	mrp-3	mrp-4	mrp-5	mrp-6	mrp-8
***B*. *ambifaria***	**95**	NS	-27	NS	NS	NS	NS	NS
***B*. *anthina***	**42**	45	50	NS	NS	58	NS	NS
***B*. *arboris***	**28**	70	72	21	NS	NS	72	72
***B*. *cenocepacia***	**64**	NS	31	NS	-45	NS	27	NS
***B*. *cepacia***	**100**	-19	NS	NS	-19	NS	-15	-18
***B*. *contaminans***	**94**	NS	NS	-17	-16	NS	NS	NS
***B*. *diffusa***	**69**	NS	NS	NS	NS	NS	NS	-26
***B*. *dolosa***	**31**	33	NS	59	NS	48	49	NS
***B*. *latens***	**42**	NS	NS	-34	NS	38	NS	-28
***B*. *metallica***	**100**	NS	NS	-13	-17	NS	NS	-10
***B*. *pseudomultivorans***	**20**	NS	NS	NS	NS	50	NS	NS
***B*. *pyrrocinia***	**70**	NS	21	-44	-35	26	NS	29
***B*. *seminalis***	**64**	NS	NS	-44	-31	NS	NS	NS
***B*. *stabilis***	**100**	-9	-47	NS	NS	-10	-63%	-77%
***B*. *ubonensis***	**52**	NS	41	NS	-24%	38	NS	32
***B*. *vietnamiensis***	**14**	NS	40	-12%	NS	57	61	35
***B*. *lata***	**18**	38	64	27	42	70	54	53
***B*. *multivorans***	**7**	33	22	21	34	62	28	45
**Strain**	**WT Mortality SKA**	**mrp-1**	**mrp-2**	**mrp-3**	**mrp-4**	**mrp-5**	**mrp-6**	**mrp-8**
***B*. *ambifaria***	**6**	61	67	30	28	75	71	58
***B*. *anthina***	**91**	9	NS	-27	-24	NS	NS	NS
***B*. *arboris***	**46**	53	54	-35	NS	NS	50	51
***B*. *cenocepacia***	**81**	NS	NS	NS	-77	-65	-24	-54
***B*. *cepacia***	**9**	81	75	NS	NS	78	62	45
***B*. *contaminans***	**47**	30	26	49	NS	NS	NS	NS
***B*. *diffusa***	**88**	NS	NS	NS	-25	NS	NS	NS
***B*. *dolosa***	**13**	NS	25	NS	NS	61	61	28
***B*. *latens***	**22**	NS	43	-19	-19	46	NS	42
***B*. *metallica***	**100**	NS	NS	-14%	-8%	NS	NS	NS
***B*. *pseudomultivorans***	**11**	NS	NS	NS	NS	30	NS	NS
***B*. *pyrrocinia***	**5**	53	61	NS	NS	64	57	46
***B*. *seminalis***	**69**	28	NS	-50	-67	NS	NS	NS
***B*. *stabilis***	**100**	-90	-58	NS	NS	-55	-60	-84
***B*. *ubonensis***	**83**	-60	NS	-28	-36	NS	NS	NS
***B*. *vietnamiensis***	**46**	-44	-39	-43	-44	-34	50	-41
***B*. *lata***	**10**	NS	NS	NS	NS	52	NS	NS
***B*. *multivorans***	**3**	NS	22	NS	NS	35	41	NS

NS = Not significant difference

**Fig 5:**
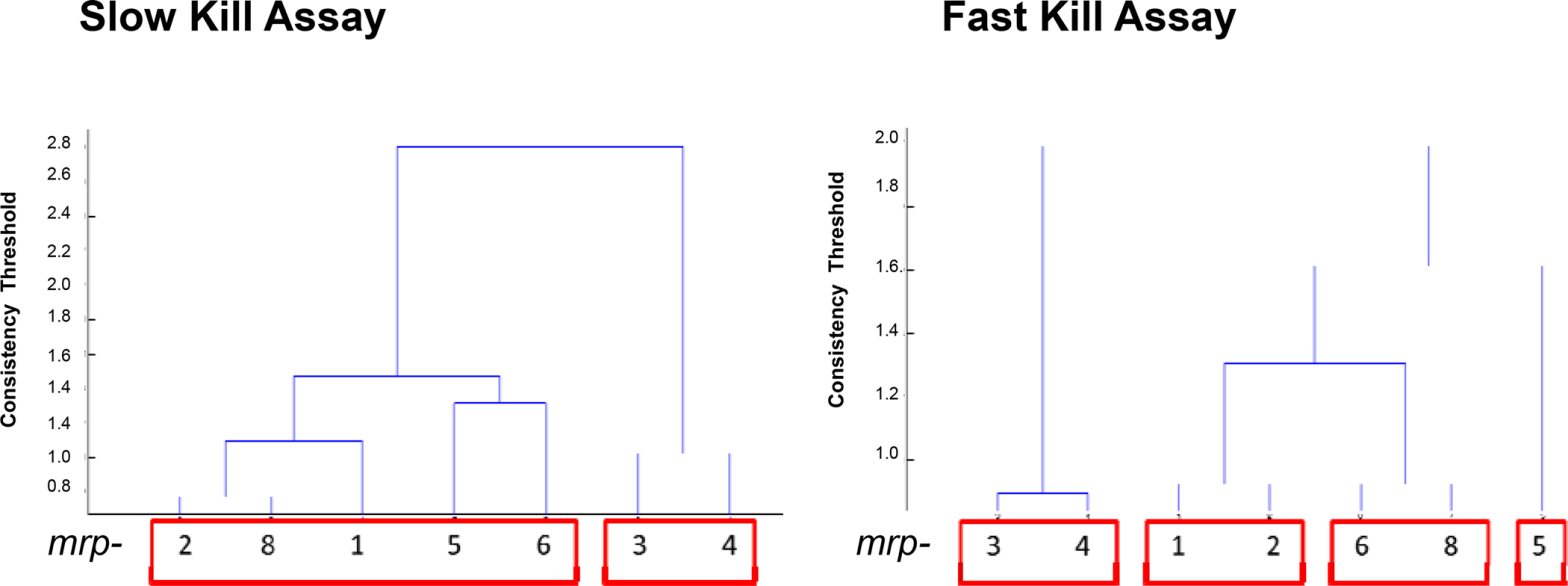
Hierarchical Clusterings. Ward’s method with a consistency threshold 1.1 used to cluster mutants based on significant changes in pathogenicity.

The complete set of killing results for each Bcc strain generated a unique killing profile in each MRP mutant. To determine whether these profiles constitute a coherent mutant classification pattern, a hierarchical clustering of significant effect sizes *vs*. each strain (Ward’s method, Consistency Threshold 1.1) was performed. This analysis showed different patterns for *mrp-3* and *mrp-4* when compared with the rest of MRP-phenotypes in both FKA and SKA. However, *mrp-3* and *mrp-4* share low sequence identity/similarity among them (data not shown), suggesting that these two transporters do not have similar substrate specificity or function. These MRP-phenotypes were grouped consistently and differentiated from the rest of single knock out strains (**Fig 5**). This pattern could justify the diverse phenotypic response to Bcc of the MRP knock-out mutants, indicating variation in substrate profile specificity for the 7 MRP efflux systems. The clustering patterns of the other transporters suggest distinct substrate specificity, in agreement with the low degree of sequence identity/similarity shared among them (data not shown). Nevertheless, we can assume that toxins and small molecules are MRP-related substrates, and these transporters play a fundamental role in Bcc defence, with the exception of *mrp-3* and *mpr-4*.

### Inhibitor experiments

The Bcc pathogenicity ranking system was used to facilitate testing for a distinct MRP-efflux system substrate profile within the content of infection. Disabling efflux pumps genetically (knock-outs) or chemically (small molecules-inhibitors) should have a similar toll on increasing nematode mortality. A pilot analysis was performed utilizing the Bcc strains that exhibited increased *C*. *elegans* susceptibility in numerous efflux knock-outs on SKA (*B*. *ambifaria*, *B*. *arboris*, *B*. *cepacia*, *B*. *dolosa*, *B*. *pyrrocinia*) and the well-characterized mammalian MRP-efflux inhibitors mometasone furoate, lasalocid A and verapamil [51]. All compounds did not affect Bcc or *C*. *elegans* viability at the concentration used for the assay, (**S1 Fig)** The compounds were spread in concentration ranges onto NGM plates to perform SKA and DMSO (0.5%) was used as a growth control. Control mortality was calculated to be the number of dead worms divided by the number of total worms. Comparison to solvent control of pooled mortality counts was done using Fisher’s Exact test; results were considered significant if Bonferroni-Holm corrected *p*-values were less than 0.05. Statistically significant differences from the WT are shown in **Table 3**. Reduction in mortality from the controls was not observed. The inhibitor use in the infection system, provided a statistically significant increase of mortality in the presence of at least one inhibitor compared to DMSO controls for 3 Bcc strains, whereas *B*. *pyrrocinia* and *B*. *ambifaria* killing rates were not affected.

**Table 3:** Effect of ABC inhibitors during *C*. *elegans*- Bcc infection on SKA. Nematodes mortality expressed as percentage of dead worms and compared between samples with inhibitors and control with DMSO (0,5%). Statistical significant differences were reported.

		Significant Changes in % Mortality								
Strain	Control Mortality	Verapamil			Mometasone			Lasalocid		
		100 μM	50 μM	25 μM	100 μM	50 μM	25 μM	500 nM	250 nM	125 nM
***B*. *cepacia***	**17**	NS	NS	NS	45	NS	NS	34	NS	NS
***B*. *arboris***	**20**	35	NS	27	45	NS	NS	NS	NS	NS
***B*. *dolosa***	**32**	NS	NS	NS	44	29	NS	48	38	NS

NS = Not significant difference

In particular, the presence of mometasone (100 μM), *B*. *arboris*, *B*. *cepacia and B*. *dolosa* enhanced virulence against nematodes with mortality rate of 40% higher than the control. Lasalocid (500 nM) caused an increase in the percentage of dead worms of 34% and 48% with *B*. *cepacia* and *B*. *dolosa*, respectively. Verapamil (100 μM) enhanced killing only for *B*. *arboris*, with a killing rate of 35% higher than the control. These results demonstrated that the inhibitor driven MRP transporter inactivation results in increased mortality at least for two inhibitors, of the nematodes to Bcc strains, supporting the role of these transporters in Bcc infection. Verapamil has been characterized as a competitive inhibitor in ABCB1 malignant cell overexpression [52] as wells as a potent ABCC family inhibitor [53]. It is also involved in inhibiting *C*. *elegans* P-gp1, which is involved in nematode resistance to ivermectine [54]. The present experimental setup differs as it explores verapamil against a number of targets simultaneously not by isolating transporters of interest. This analysis suggests that verapamil very likely works, but the number of interactions leading to a weaker phenotype should be investigated further.

## Conclusions

**The major aim of this work** was to inquire the role of host transporters in the infection developing a nematode virulence ranking system focusing in well-recognized Bcc strains. It is common knowledge that every bacterial species includes member-strains with different pathogenic characteristics. However, the key purpose was to build the model using type strains of each of the 18 currently known Bcc species, a combination of two different killing assays (SKA and FKA), and a set of nematode mutants impaired in MRP efflux transporters. We focused on type strains due to the extensive information known, as many Bcc genomes have been completely sequenced and more will be soon become available. To define Bcc virulence we established a VR scheme, based on the percentage of surviving worms. Both nematocidal assays revealed different pathogenicity profiles for the Bcc species. Strains with high score in the VR system were able to accumulate in the nematodes intestine and produce virulence factors, on SKA and FKA, respectively. Only Bcc CF isolates accumulate within worms, an observation that correlates well with the apparent differences in virulence factors between environmental and CF isolates. This VR scheme was applied to profile Bcc pathogenesis in seven MRP impaired *C*. *elegans* mutants. MRPs are implicated in distinct nematode cellular processes: MRP1 is involved in heavy metal tolerance and ivermectine resistance [42,43]; MRP4 is central in early-stage differentiation [55]; MRP5 acts as a fundamental heme exporter into embryonic development [56]. Results showed increased nematode mortality for several *C*. *elegans* mutants grown in the presence of specific Bcc strains compared to WT nematodes. In particular *mrp-2* and *mrp-5* were the most susceptible mutants with increased mortality respectively in 13 and 11 different Bcc strains in the two assays, suggesting an active role of these two efflux transporters in host defense. However strain mpr-5 was tested only in heterozygosis and this could have affected survival rate. Cluster analysis consistently grouped and separated *mrp-3* and *mrp-4* mutants in both assays from the other MRP-phenotypes. This pattern suggested different substrate specificity for these MRP transporters. To further explore the role of MRP transporters in host defense, inhibitor experiments were carried out on a selected panel of the five most "infectious" Bcc strains against the MRP knock-out mutants (*B*. *ambifaria*, *B*. *arboris*, *B*. *cepacia*, *B*. *dolosa*, *B*. *pyrrocinia)*. These strains were tested against the WT nematodes in the presence of three well-characterized MRP-inhibitors, with a broad inhibitory activity against MRP transporters [51]. These results suggested that chemically disabling of the MRPs resulted in increased *C*. *elegans* susceptibility to Bcc strains. The use of mometasone and lasalocid in the infection system increased killing rate when incubated with *B*. *cepacia* and *B*. *dolosa*, while verapamil showed a mild effect for *B*. *arboris*.

In conclusion, this study provided tools to correlate microbial pathogenicity with the host transporters, and highlighted specific efflux systems with a central role in Bcc virulence.

It is worth noting that MRPs share components of a conserved activation mechanism with the Cystic Fibrosis Transmembrane conductance Regulator (CFTR)[57,58]. Therefore, identification of bacterial signalling molecules with substrate specificity in recognizing MRPs CFRT-like efflux transporters involved in host response could be a starting point for the development of novel therapeutic strategies.

## Supporting Information

S1 Fig Bcc strain growth in the presence of inhibitors. For the Bcc strain growth curves in the presence of inhibitors, one single colony of each Bcc strain was placed in a tube containing 3 mL of LB broth. The tubes were then incubated at 37°C overnight in agitation. The overnight cultures were used to inoculate 250 mL flasks containing 50 mL of LB broth plus the inhibitors at an initial concentration of 0.01 OD600/mL. For each strain, a set of 5 flasks was employed: 1) Verapamile 100 μM; 2) Mometasone 100 μM; 3) Lasalocid 500 nM; 4) DMSO 0.5% v/v; 5) control (no inhibitor or DMSO). The flasks were incubated at 37°C in agitation at 220 rpm. Bacterial growth was monitored following OD600 for 36 hours every 2 hours. The experiments were performed in duplicate and the data reported represent mean values. Error bars were omitted for clarity. Results proved that the inhibitors did not interfere with Bcc growth at the concentration used in our assays, as the growth curves obtained for each strain are very similar with no viable effect in growth by any inhibitor.
(DOC)
