# Supplementary material for: Investigating the Role of the Host Multidrug Resistance Associated Protein Transporter Family in Burkholderia cepacia Complex Pathogenicity Using a Caenorhabditis elegans Infection Model
Source: PLoS One. 2015 Nov 20;10(11):e0142883. doi: 10.1371/journal.pone.0142883 (PMC4654563; doi:10.1371/journal.pone.0142883)

**S1 Fig. Bcc strain growth in the presence of inhibitors.**

For the Bcc strain growth curves in the presence of inhibitors, one single colony of each Bcc strain was placed in a tube containing 3 mL of LB broth. The tubes were then incubated at 37°C overnight in agitation. The overnight cultures were used to inoculate 250 mL flasks containing 50 mL of LB broth plus the inhibitors at an initial concentration of 0.01 OD_600_/mL. For each strain, a set of 5 flasks was employed: 1) Verapamile 100 μM; 2) Mometasone 100 μM; 3) Lasalocid 500 nM; 4) DMSO 0.5% v/v; 5) control (no inhibitor or DMSO). The flasks were incubated at 37°C in agitation at 220 rpm.

Bacterial growth was monitored following OD_600_ for 36 hours every 2 hours. The experiments were performed in duplicate and the data reported represent mean values. Error bars were omitted for clarity.

Results proved that the inhibitors did not interfere with Bcc growth at the concentration used in our assays. as The growth curves obtained for each strain are very similar with no viable effect in growth by any inhibitor.


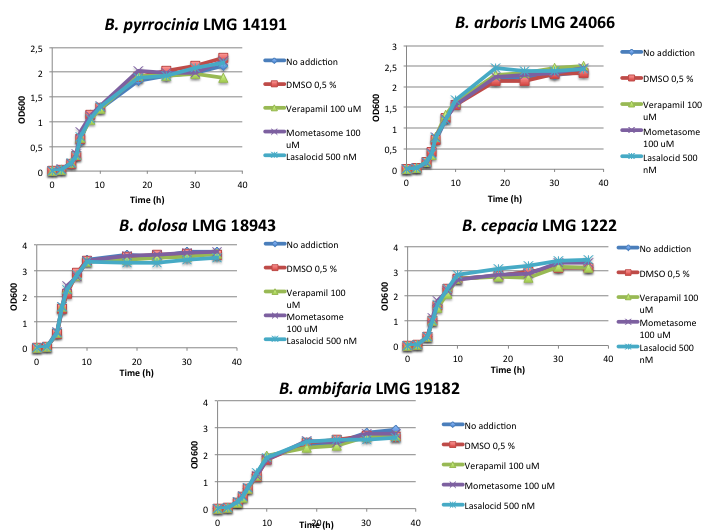

Supplement: S1 Fig — For the Bcc strain growth curves in the presence of inhibitors, one single colony of each Bcc strain was placed in a tube containing 3 mL of LB broth. The tubes were then incubated at 37°C overnight in agitation. The overnight cultures were used to inoculate 250 mL flasks containing 50 mL of LB broth plus the inhibitors at an initial concentration of 0.01 OD600/mL. For each strain, a set of 5 flasks was employed: 1) Verapamile 100 μM; 2) Mometasone 100 μM; 3) Lasalocid 500 nM; 4) DMSO 0.5% v/v; 5) control (no inhibitor or DMSO). The flasks were incubated at 37°C in agitation at 220 rpm. Bacterial growth was monitored following OD600 for 36 hours every 2 hours. The experiments were performed in duplicate and the data reported represent mean values. Error bars were omitted for clarity. Results proved that the inhibitors did not interfere with Bcc growth at the concentration used in our assays, as the growth curves obtained for each strain are very similar with no viable effect in growth by any inhibitor. (DOC) [file pone.0142883.s001.doc]
